# Targeting the microbiota-gut-brain axis in post-stroke insomnia: a phase-dependent therapeutic framework

**DOI:** 10.3389/fnins.2026.1784251

**Published:** 2026-04-09

**Authors:** Yumin Zhou, Yingying Huang, Xia Bi

**Affiliations:** Shanghai University of Medicine and Health Sciences Affiliated Zhoupu Hospital, Shanghai, China

**Keywords:** glymphatic system, microbiota-gut-brain axis, NLRP3 inflammasome, post-stroke insomnia, precision medicine, tryptophan metabolism, washed microbiota transplantation

## Abstract

Post-stroke insomnia (PSI) is a critical biological barrier to neurorehabilitation afflicting over half of all stroke survivors. Traditional sedatives often force clinicians into a therapeutic dilemma between sleep efficacy and cognitive suppression. The microbiota-gut-brain (MGB) axis has recently emerged as a transformative target to resolve this impasse. Acute stroke triggers profound autonomic dysfunction, causing immediate intestinal barrier collapse. This “leaky gut” facilitates the systemic translocation of lipopolysaccharides (LPS) and activates the NLRP3 inflammasome. The resulting inflammatory storm hijacks central tryptophan metabolism via the indoleamine 2,3-dioxygenase (IDO) enzyme. This “tryptophan steal” diverts serotonin precursors toward neurotoxic kynurenine pathways, driving severe cortical hyperarousal. Sleep fragmentation then prevents the glymphatic system from clearing metabolic waste, further exacerbating neuroinflammation. To break this vicious cycle of neurotoxicity, we propose a phase-dependent therapeutic framework. During the highly vulnerable acute phase, interventions must prioritize gut barrier protection using postbiotics to mitigate infection risks under CNS injury-induced immunodepression (CIDS), often discussed as stroke-induced immunosuppression. As patients enter the chronic phase, therapy shifts toward metabolic restoration using live therapeutics, such as washed microbiota transplantation (WMT) and next-generation psychobiotics like *Akkermansia muciniphila*. Targeting the MGB axis offers a mechanism-based strategy to achieve precision sleep medicine, restoring the biological foundation necessary for optimal neuroplasticity and recovery.

## 1 Introduction

### 1.1 The silent epidemic: beyond symptomology

Stroke remains a formidable global health challenge, consistently ranking among the leading causes of mortality and long-term adult disability worldwide (GBD 2019 Stroke Collaborators, 2021; Feigin et al., 2025). While the advent of hyper-acute recanalization therapies—such as mechanical thrombectomy and intravenous thrombolysis—has improved acute outcomes, these advances have coincided with a growing population of survivors living with chronic sequelae (GBD 2019 Stroke Collaborators, 2021; Feigin et al., 2025). Among these, sleep–wake disturbances are pervasive yet frequently underestimated in routine clinical practice, often overshadowed by more visible motor deficits (Khot and Morgenstern, 2019). Epidemiological evidence suggests that sleep problems affect roughly half of stroke survivors, consistent with pooled estimates of poor sleep quality after stroke and broader post-stroke sleep-disorder burden (Khot and Morgenstern, 2019; Luo et al., 2023). Of these disorders, post-stroke insomnia (PSI) is commonly reported and can persist, presenting a barrier to effective neurorehabilitation (Wang et al., 2024; Sun et al., 2026). PSI is characterized not merely by difficulties in sleep initiation, but also by fragmentation of sleep continuity and non-restorative rest (Wang et al., 2024). The clinical ramifications of untreated PSI extend beyond subjective fatigue or daytime somnolence; disrupted sleep after stroke has been associated with poorer functional outcomes and increased risk of adverse vascular events in observational and review evidence (Khot and Morgenstern, 2019). Sleep is a fundamental physiological pillar for neuroplasticity and memory consolidation, and sleep disruption may therefore hinder recovery processes after stroke (Khot and Morgenstern, 2019). Indeed, disrupted sleep in the post-stroke period has been linked to worse functional recovery and mood and cognitive outcomes, supporting proactive recognition and targeted management rather than passive observation.

### 1.2 The Therapeutic dilemma: limitations of current pharmacotherapy

Despite the clear imperative to manage sleep effectively, clinicians often face a therapeutic dilemma because current pharmacological options remain suboptimal. The hypnotic landscape is still dominated by GABAergic sedatives, mainly benzodiazepines and Z-drugs, which can be effective for short-term sedation but carry clinically important safety trade-offs in older inpatients (Aoki et al., 2025). In older adults hospitalized with acute ischemic stroke, initiating benzodiazepines within 3 days was associated with a higher 10-day risk of falls or fall-related injuries compared with non-initiation, underscoring the need for caution during early recovery when patients may be mobilizing (Sun et al., 2022). More critically, converging preclinical evidence indicates that heightened GABAergic inhibition in peri-infarct cortex can constrain post-stroke plasticity, and that reducing this excessive tonic inhibition can improve functional recovery (Clarkson et al., 2010). Consistent with this mechanistic concern, experimental work cited in a mouse focal cortical stroke model reports that administering a benzodiazepine after rehabilitation can produce an acute loss of regained function, aligning with the concept that enhancing GABAergic signaling may counteract relearning-dependent recovery (Alia et al., 2016). This creates an impasse: clinicians must balance the need for restorative sleep against the risk of drug-associated falls and the possibility of blunting experience-dependent recovery processes (Alia et al., 2016; Sun S. et al., 2025). The conundrum underscores an unmet need for novel interventions that are safer in the early rehabilitation window and that target pathophysiological drivers of insomnia rather than only suppressing arousal (Dressle et al., 2025).

### 1.3 The MGB axis: a paradigm shift

Building upon the foundational framework established by seminal works linking the gut microbiota to neurobehavioral and neuroendocrine regulation (Clarke et al., 2014; Cryan et al., 2019), the microbiota-gut-brain (MGB) axis has emerged as a central paradigm. It is now crucial for understanding how peripheral organ systems, particularly the gastrointestinal tract, influence recovery and complications after ischemic stroke (Macom and Brown, 2025; Shen et al., 2025). After ischemic stroke, intestinal barrier integrity can be compromised and intestinal permeability can increase, with downstream consequences including bacterial translocation and systemic inflammation. Concurrently, post-stroke gut dysbiosis is commonly characterized by a reduction in beneficial taxa and metabolites (including short-chain fatty acids, SCFAs), alongside an expansion of potentially pathogenic bacteria that may promote endotoxemia and immune activation (Macom and Brown, 2025; Shen et al., 2025). In parallel, inflammasome-related inflammatory pathways appear to be engaged after stroke in both the gut and the brain, and experimental post-stroke models link dysbiosis-driven barrier disruption and systemic inflammation to microglial NLRP3 inflammasome activation (Chen et al., 2025; Macom and Brown, 2025). Crucially, dysbiosis may contribute to neuropsychiatric sequelae through immune–metabolic reprogramming rather than acting as a passive bystander. Inflammation after ischemic stroke has been linked to increased tryptophan catabolism through the kynurenine pathway (higher KYN/TRP ratios), which correlates with stroke severity and long-term outcome in clinical observations (Brouns et al., 2010; Guido et al., 2022). Because kynurenine-pathway metabolites can be neuroactive, shifts toward neurotoxic products (e.g., quinolinic acid) have been proposed as one mechanism by which systemic inflammation could amplify neuroinflammation and excitotoxic stress relevant to sleep–wake dysregulation (Yao et al., 2025). Once sleep fragmentation develops, experimental evidence shows it can directly suppress glymphatic clearance and reduce interstitial waste removal, providing a plausible feedback loop in which disturbed sleep further sustains neuroinflammatory stress (Deng et al., 2024). Consequently, this review hypothesizes that effective management of post-stroke insomnia may benefit from moving beyond symptom-based sedation toward mechanism-based strategies that also target gut barrier function and microbiota-linked inflammatory–metabolic pathways.

## 2 The clinical and pathophysiological landscape

### 2.1 Phenomenology and altered sleep architecture

Post-stroke sleep disorders encompass a broad spectrum, including sleep-disordered breathing, restless legs/periodic limb movements, and circadian rhythm disruption (Baglioni et al., 2016). However, insomnia is a particularly common non-respiratory complaint and may persist well beyond the acute event (Yang et al., 2024). Distinct from primary insomnia, post-stroke insomnia (PSI) often presents with difficulty maintaining sleep (sleep fragmentation), frequent awakenings, and early morning awakening (Yang et al., 2024). This pattern suggests disruption in mechanisms that sustain sleep continuity after brain injury (Baglioni et al., 2016). Objective verification via polysomnography (PSG) supports clinically meaningful changes in sleep continuity after stroke, including lower sleep efficiency and greater wake after sleep onset (WASO) relative to control populations (Baglioni et al., 2016). PSG meta-analytic data also suggest reduced slow-wave sleep (SWS) after stroke, while findings for REM sleep are more heterogeneous across studies and settings (Baglioni et al., 2016). SWS (N3) is tightly linked to restorative physiology, including enhanced glymphatic clearance during slow-wave sleep, whereas REM sleep has been implicated in emotional brain function (Goldstein and Walker, 2014; Reddy and van der Werf, 2020). Sleep also supports motor skill learning and procedural memory consolidation—processes directly relevant to post-stroke motor relearning—through sleep-dependent network reorganization that has been demonstrated in human procedural-learning experiments (Vahdat et al., 2017). Consistent with this mechanistic rationale, selective loss of restorative sleep features in PSI may plausibly contribute to slower functional gains during rehabilitation, and several stroke PSG studies have examined links between sleep-stage metrics and functional outcomes (e.g., Barthel Index) (Baglioni et al., 2016).

### 2.2 The glymphatic connection: the brain’s waste management

One of the strongest reasons to prioritize sleep in stroke recovery is the glymphatic system, a perivascular waste-clearance pathway that depends on astroglial aquaporin-4 (AQP4) water channels and is most active during sleep (Rasmussen et al., 2018). While direct evidence in stroke cohorts is pending, fundamental rodent experiments demonstrate that natural sleep (and anesthesia) is associated with a >60% expansion of interstitial space volume fraction, which facilitates cerebrospinal fluid (CSF)–interstitial fluid exchange and accelerates clearance of metabolites including β-amyloid (Xie et al., 2013). Stroke injury can impair glymphatic function through peri-infarct astrogliosis and loss of perivascular AQP4 polarization, changes that have been observed in experimental cerebral ischemia models (Sun et al., 2022). When superimposed with PSI and slow-wave sleep (SWS) loss, the brain may be deprived of an important “cleaning cycle” that supports metabolic homeostasis during recovery (Xie et al., 2013; Rasmussen et al., 2018). The resulting retention of neurotoxic proteins (e.g., amyloid-β and tau) and inflammatory mediators may plausibly worsen the biochemical milieu for repair and contribute to secondary neurodegenerative processes (Rasmussen et al., 2018, p. 2022; Sun et al., 2022). Thus, treating insomnia after stroke is not only about comfort; it may also help preserve sleep-dependent waste clearance mechanisms that could be relevant to neurological recovery (Xie et al., 2013; Sun et al., 2022).

### 2.3 The bidirectional risk architecture and pre-existing vulnerabilities

The relationship between stroke and sleep is fundamentally bidirectional, and it creates reciprocal pathways of risk and consequence (Rissardo et al., 2025). Pre-existing sleep disorders increase the risk of incident stroke, and mechanistic links include autonomic dysregulation (including heightened sympathetic activity), systemic inflammation, endothelial dysfunction, and blood pressure variability (Rissardo et al., 2025). In parallel, genetic epidemiology supports causality: a Mendelian randomization study linked genetic predisposition to insomnia with higher ischemic stroke risk, with signals in large artery atherosclerosis and small vessel stroke (Li et al., 2025). This risk architecture often unfolds in patients who already carry vascular comorbidities such as hypertension, atherosclerosis, and metabolic dysfunction, which overlap biologically with inflammatory and endothelial pathways implicated in sleep-related cerebrovascular risk (Rissardo et al., 2025). These same vascular and metabolic phenotypes are also linked to gut microbiome disruption through immune and metabolic mechanisms that can promote chronic, low-grade inflammatory states (Lin et al., 2024). Therefore, when an acute cerebrovascular event occurs, it may act as a “second hit” that amplifies pre-existing vulnerability rather than initiating gut ecosystem disruption from a pristine baseline (Lin et al., 2024). A history of pre-stroke sleep disturbance may further compound this vulnerability because sleep disruption can alter gut microbial structure and function through circadian, immune, and metabolite-mediated pathways (Lin et al., 2024). Consistent with this, insomnia risk and its correlates are clinically measurable in the ischemic stroke convalescence period, underscoring that sleep pathology is common and clinically structured in this population (Sun X. et al., 2025). As a result, the dysbiosis observed in post-stroke insomnia could reflect exacerbation of baseline microbiome dysfunction rather than a purely *de novo* phenomenon (Lin et al., 2024). Future large-scale clinical studies should therefore include matched non-stroke controls with similar sleep disorders and vascular risk profiles. This design is needed to distinguish stroke-specific microbial signatures from changes driven by pre-existing sleep and cardiometabolic disease.

## 3 Pathophysiological mechanisms: anatomy of the vicious cycle

**FIGURE 1:**
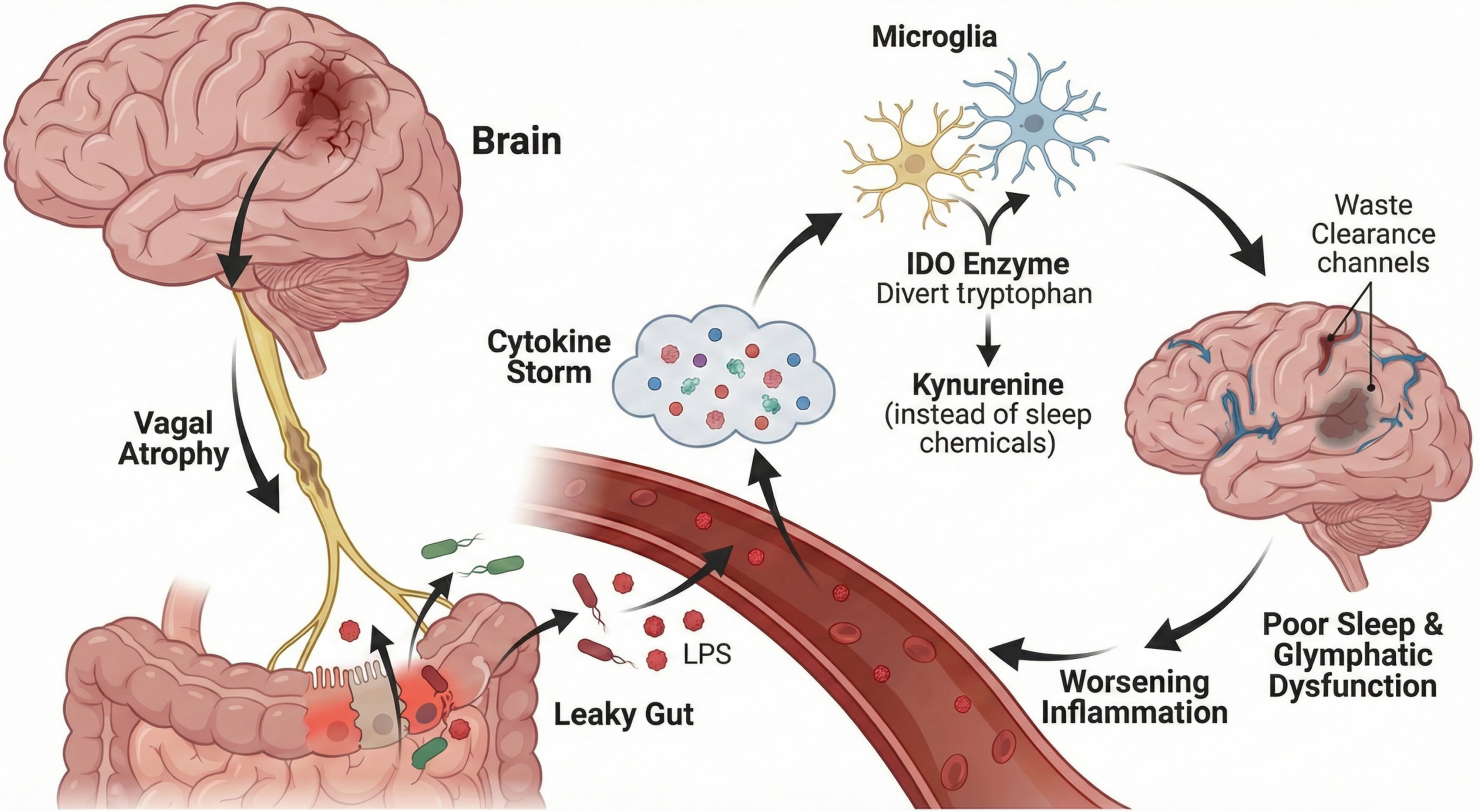
The vicious cycle of the microbiota-gut-brain axis in post-stroke insomnia (PSl). Stroke-induced neural injury, particularly affecting the vagus nerve (vagal withdrawal), compromises gastrointestinal motility. This autonomic failure causes a “leaky gut,” allowing luminal bacteria and endotoxins (LPS) to translocate into the systemic circulation. This triggers a systemic “cytokine storm” that reaches the brain, activating microglia and the IDO enzyme. IDO diverts tryptophan metabolism toward neurotoxic kynurenine production rather than sleep-promoting neurotransmitters (e.g., serotonin). The resulting sleep fragmentation prevents metabolic waste clearance via the glymphatic system, further exacerbating neuroinflammation and perpetuating the cycle. (PSI, post-stroke insomnia; LPS, lipopolysaccharide; IDO, indoleamine 2,3-dioxygenase).

The mechanistic coupling between gut dysbiosis and PSI is not linear but circular. It involves synergistic interactions that form a self-perpetuating “vicious cycle” of pathology (Figure 1). To fully understand this mechanism, it must be viewed through two mutually reinforcing pathways.

### 3.1 The “top-down” pathway (brain injury to leaky gut)

The cycle can begin with the acute cerebrovascular event, which perturbs brain–gut communication through autonomic and inflammatory signaling (Arya and Hu, 2018). Experimental evidence indicates that gut barrier permeability can rise within hours after stroke, and this effect is mediated in part by sympathetic nervous system activation that drives intestinal epithelial injury (Prame Kumar et al., 2025). In this context, the early “leaky gut” state creates conditions that can accelerate microbial dysbiosis and increase the likelihood of pathobiont expansion and translocation (Arya and Hu, 2018; Prame Kumar et al., 2025).

### 3.2 The “bottom-up” pathway (leaky gut to hyperarousal)

When barrier integrity is compromised, intestinal luminal bacteria and toxins can enter host tissues and circulation, amplifying systemic inflammation (including severe systemic inflammatory responses in some settings) (Arya and Hu, 2018). A key candidate mediator is lipopolysaccharide (LPS), which can drive inflammatory signaling and microglial activation in experimental systems (Sheppard et al., 2019). In experimental brain tissue models, LPS can activate microglia and increase microglia-derived IL-1β, with downstream synaptic disruption—mechanistic steps that are consistent with a pathway linking peripheral inflammatory cues to central dysfunction (Sheppard et al., 2019). Together, these pathways can form a self-reinforcing loop: stroke-associated gut permeability and dysbiosis can shape neuroinflammatory responses after stroke, and this inflammatory state may contribute to sustained cortical hyperarousal and poor sleep (Arya and Hu, 2018). Prior work supports the principle that post-stroke microbiota states can influence neuroinflammation, consistent with a closed pathogenic loop that warrants targeted efforts to interrupt brain–gut feedback (Singh et al., 2016; Arya and Hu, 2018).

### 3.3 The immune-inflammatory cascade: TLR4 and NLRP3 activation

The immune pathway provides a plausible mechanistic bridge between impaired intestinal barrier function and central neuroinflammation (Ma et al., 2024; Zhang et al., 2024). Following acute stroke, systemic stress pathways are activated, including sympathetic–adrenal and HPA-axis signaling, with catecholamines and glucocorticoids as key mediators (Arya and Hu, 2018; Molotla-Torres et al., 2023). Experimental and stress-related models suggest that stress hormones can remodel epithelial tight junctions and increase paracellular permeability, partly through changes in tight junction protein expression and/or localization (Molotla-Torres et al., 2023). Consistent with this, glucocorticoid-associated stress paradigms have been linked to reduced levels of tight junction proteins (e.g., claudin-1, occludin, and ZO-1), which aligns with a shift toward a “leaky gut” phenotype (Molotla-Torres et al., 2023). When barrier function is compromised after stroke, bacterial products may translocate beyond the intestinal lumen and contribute to systemic immune activation (Crapser et al., 2016; Stanley et al., 2016). In preclinical murine models, stroke-associated gut barrier dysfunction has been reported to precede dissemination of orally inoculated bacteria to peripheral sites, supporting a temporal link from barrier impairment to systemic exposure in some settings (Stanley et al., 2016). These conditions can increase the likelihood of endotoxemia, in which LPS enters the circulation and amplifies inflammatory signaling (Sheppard et al., 2019). LPS binds Toll-like receptor 4 (TLR4) and can activate downstream programmes such as NF-κB, promoting transcription of pro-inflammatory mediators (Sheppard et al., 2019; Arya et al., 2025). TLR4-linked inflammatory signaling can also support inflammasome activation (Paik et al., 2021). In cerebral ischemia/reperfusion models, increased TLR4 signaling has been associated with higher NLRP3 expression and inflammatory mediator levels, whereas TLR4 knockdown reduced NLRP3-related responses (Mao et al., 2023). Once inflammasome pathways are engaged, caspase-1-dependent processing of pro-IL-1β and pro-IL-18 yields active IL-1β and IL-18, which can contribute to a broader systemic inflammatory response with elevated circulating cytokines (Paik et al., 2021). Peripheral cytokines can communicate with the brain through several routes, including interactions at the blood–brain barrier, neural afferent pathways (including vagal signaling), and circumventricular structures (Arya and Hu, 2018; Ma et al., 2024). Within the CNS, these signals may promote microglial activation and sustain a neuroinflammatory milieu that can alter neuronal excitability in sleep-regulating circuits (Sheppard et al., 2019; Ma et al., 2024). Cytokines such as IL-1β and TNF-α are established modulators of non-REM sleep physiology, and elevated inflammatory tone has been linked to altered sleep architecture and sleep fragmentation in experimental settings (Krueger, 2008; Kaushal et al., 2012).

### 3.4 Metabolic mediators: the “tryptophan steal” and kynurenine toxicity

While the immune cascade sets the stage, the metabolic consequences of inflammation can also alter sleep-relevant neurochemistry. This process centers on tryptophan, an essential amino acid and a precursor for serotonin (5-HT) and melatonin (Qin et al., 2025). In a healthy gut, commensal microbes and their metabolites can promote enterochromaffin-cell serotonergic output (e.g., via effects on TPH1 expression), thereby shaping peripheral 5-HT biology (Reigstad et al., 2015; Legan et al., 2022). Although peripheral serotonin does not cross the blood–brain barrier, tryptophan does cross via competitive transport, and peripheral handling of tryptophan can therefore influence central substrate availability (Legan et al., 2022). However, a pro-inflammatory post-stroke milieu can shift tryptophan metabolism toward the kynurenine pathway (Qin et al., 2025). Pro-inflammatory cytokine signaling can induce IDO, driving tryptophan catabolism into kynurenine derivatives and can reduce tryptophan availability for serotonin synthesis (Soichot et al., 2011). This inflammation-associated diversion is often described as a tryptophan “steal” or “switch” (Soichot et al., 2011; Qin et al., 2025). This diversion may affect the CNS through two coupled mechanisms (Qin et al., 2025). First, reduced tryptophan availability for serotonin/melatonin synthesis may weaken sleep- and circadian-relevant signaling. Second, increased kynurenine-pathway activity can raise levels of neuroactive metabolites, including 3-hydroxykynurenine and quinolinic acid (QUIN) (Pérez-De La Cruz et al., 2012). QUIN is an endogenous NMDA receptor agonist, and its toxicity has been linked to excitotoxic and oxidative mechanisms under pathological conditions (Pérez-De La Cruz et al., 2012). Together, reduced serotonin/melatonin-related capacity and increased neuroactive kynurenines may contribute to hyperarousal and sleep fragmentation, and they may also help explain why approaches focused only on increasing serotonin can be insufficient if upstream inflammatory diversion persists (Qin et al., 2025).

### 3.5 Neuroendocrine dysregulation: the HPA axis loop

The hypothalamic–pituitary–adrenal (HPA) axis is a core neuroendocrine stress system and is closely coupled to sleep–wake regulation. An acute cerebrovascular event is likely to engage this axis, progressing from hypothalamic corticotropin-releasing hormone (CRH) to pituitary adrenocorticotropic hormone (ACTH) and then adrenal cortisol, with negative-feedback control involving higher brain regions including the hippocampus (Balbo et al., 2010). Sleep and HPA activity interact bidirectionally. Sleep onset tends to inhibit cortisol secretion, whereas awakenings are accompanied by transient cortisol elevations, and low cortisol levels are temporally associated with greater slow-wave sleep (SWS) (Balbo et al., 2010). Beyond cortisol itself, elevated CRH tone is linked to higher-frequency sleep EEG activity with reduced SWS and more wakefulness, providing a plausible route by which sustained HPA activation could contribute to longer sleep latency and sleep maintenance problems in the post-stroke period (Balbo et al., 2010). In the post-stroke context, HPA dysregulation may also be shaped by the gut–brain axis. Preclinical evidence from germ-free and colonization models indicates that gut microbes can calibrate HPA-axis stress responsivity, and that absence of microbiota is associated with an exaggerated HPA response that can be partially normalized by microbial colonization during early life (Rosin et al., 2021). This literature supports the broader premise that microbiome disruption could reduce the stability of neuroendocrine stress regulation, thereby sustaining arousal signals that are adverse for restorative sleep (Balbo et al., 2010; Rosin et al., 2021).

### 3.6 The neural pathway: vagal atrophy and the cholinergic blockade

The vagus nerve is a major bidirectional conduit of the microbiota–gut–brain axis, carrying “bottom-up” visceral afferent information to the brainstem and contributing to “top-down” regulation of gut function and inflammation (Arya and Hu, 2018; Lin et al., 2024). After stroke, autonomic imbalance can include relatively increased sympathetic drive with reduced parasympathetic (vagal) tone, which is linked to gastrointestinal dysmotility and may contribute to constipation, intestinal stasis, and conditions favoring bacterial overgrowth (Arya and Hu, 2018; Zhang et al., 2024). Mechanistically, reduced vagal afferent signaling can attenuate gut-to-brain communication: microbial metabolites and gut-derived neuroactive signals (including serotonin-related pathways and short-chain fatty acids) are discussed as inputs that may engage vagal afferents and brainstem integration (including the nucleus tractus solitarius) with downstream effects on arousal and sleep regulation (Lin et al., 2024). In parallel, the vagus nerve is central to the cholinergic anti-inflammatory pathway, and reduced vagal activity may weaken this inhibitory control over peripheral inflammation, potentially sustaining systemic inflammatory signaling that can propagate neuroinflammation and worsen sleep disruption (Arya and Hu, 2018; Zhang et al., 2024).

## 4 Gut microbiota dysbiosis: the microbial signature of insomnia

Analyses of the gut microbiome in stroke cohorts indicate that patients who go on to develop post-stroke sleep disorders show a distinguishable microbial profile compared with those without sleep disorders, supporting the concept of a sleep-related “microbial signature” in the post-stroke setting (Xie et al., 2023). More broadly, ischemic stroke has been repeatedly associated with gut dysbiosis featuring loss of commensal taxa and enrichment of opportunistic organisms, which provides a biological context in which sleep outcomes may be shaped by the intestinal ecosystem rather than merely tracked by it (Sun et al., 2021; Zhang et al., 2024).

### 4.1 Taxonomic shifts and diversity loss

A consistent observation in post-stroke sleep disorder cohorts is reduced alpha-diversity (particularly evenness), alongside community-level restructuring, which is consistent with a less stable microbial ecosystem (Xie et al., 2023). In that prospective study, post-stroke sleep disorders were also associated with an enterotype distribution shift toward an *Escherichia*–*Shigella*-dominated community, suggesting a pattern that is not purely stochastic (Xie et al., 2023). Taxonomically, these profiles often show depletion of SCFA-associated commensals and enrichment of facultative anaerobes/opportunists. In the post-stroke sleep disorder cohort, relative abundances of several putatively beneficial genera (including *Bifidobacterium*, *Lactobacillus*, and *Faecalibacterium*) were lower in the sleep-disorder group, while LEfSe highlighted *Streptococcus* and *Blautia* among key discriminatory signals for classification (Xie et al., 2023). Complementary stroke literature also reports that worse clinical phenotypes can co-occur with reduced alpha-diversity and depletion of SCFA-producing bacteria (including *Faecalibacterium* and *Roseburia*), consistent with reduced SCFA-related support for barrier integrity and neuroimmune homeostasis (Sun et al., 2021). Conversely, enrichment of opportunistic taxa after stroke frequently involves Proteobacteria-associated lineages and Enterobacteriaceae-linked genera in broader stroke dysbiosis patterns, which plausibly increases exposure to pro-inflammatory microbial products; in this context, lipopolysaccharide (LPS) in this context, LPS remains relevant to the inflammatory pathways described earlier (Sun et al., 2021; Arya et al., 2025).

### 4.2 Functional dysbiosis: the metabolic void

Beyond taxonomic abundance, post-stroke dysbiosis is also associated with disrupted microbial function, including reduced capacity for fiber fermentation and downstream SCFA production (Zhang et al., 2024). SCFAs (notably butyrate, propionate, and acetate) are increasingly recognized as signaling metabolites with immunometabolic effects relevant to neurovascular integrity (Fock and Parnova, 2023; Lin et al., 2024). Mechanistically, SCFAs have been linked to blood–brain barrier protection and to regulation of neuroinflammatory pathways, providing a plausible route by which SCFA depletion could remove counter-regulatory constraints on post-stroke neuroinflammation (Fock and Parnova, 2023; Lin et al., 2024). Functional dysbiosis may also reduce microbiota-derived neuroactive signaling. Several gut taxa are discussed as contributors to neuroactive compound production (including GABA, in a strain- and context-dependent manner), and reduced representation of these functional guilds could weaken inhibitory signaling relevant to nocturnal arousal control (Lin et al., 2024). Taken together—loss of SCFA-associated functions, enrichment of pro-inflammatory pathobionts, and disruption of neuroactive-metabolite pathways—these changes can be framed not only as diagnostic correlates but also as candidate, modifiable therapeutic targets (Lin et al., 2024; Zhang et al., 2024). This logic supports moving beyond generic supplementation toward more precise strategies that aim to restore specific functional capacities (e.g., SCFA-producing networks and neuroactive-metabolite pathways) while limiting pro-inflammatory overgrowth (Zhang et al., 2024; Table 1).

**TABLE 1:** Key microbial alterations in post-stroke insomnia and their physiological effects.

Microbial taxon	Trend in PSI	Key metabolite	Target pathway	Physiological mechanism (gut-brain link)	Sleep architecture impact	Key references
Butyrate Producers (e.g., *Faecalibacterium prausnitzii, Roseburia*)	↓↓	Butyrate (SCFA)	NF-κB/HDAC	Loss of butyrate reduces HDAC inhibition, leading to unchecked systemic inflammation (IL-6, TNF-α) and BBB permeability.	Deep sleep loss: significant reduction in Slow-Wave Sleep (SWS) duration and intensity.	Sun et al., 2021; Fock and Parnova, 2023
GABA/serotonin producers (e.g., *Bifidobacterium*, *Lactobacillus*)	↓	GABA, tryptophan	HPA axis/vagus	Reduced precursors for inhibitory neurotransmitters remove the “brake” on the HPA axis, leading to hypercortisolemia.	Sleep latency: difficulty falling asleep; increased anxiety-related arousal.	Xie et al., 2023; Lin et al., 2024
Opportunistic pathogens (e.g., Enterobacteriaceae, *Escherichia, Shigella*)	↑↑	LPS (endotoxin)	TLR4/NLRP3	LPS translocation triggers a cytokine storm via NLRP3 inflammasome activation; promotes IDO-mediated tryptophan depletion.	Fragmentation: increased wake after sleep onset (WASO); “sickness behavior” sleep.	Xie et al., 2023; Sheppard et al., 2019
Oral-gut translocators (e.g., *Streptococcus*)	↑	Pro-inflammatory cytokines	Immune system	Translocation due to dysphagia/CIDS correlates with stroke severity; drives systemic inflammatory load.	Efficiency: reduced overall sleep efficiency and quality.	Xie et al., 2023
Visceral sensitivity modulators (e.g., *Blautia*)	↓	Gas/metabolites	ENS afferents	Loss of these bacteria dysregulates visceral sensation, leading to hypersensitivity and sub-threshold pain signaling.	Nocturnal arousal: frequent micro-arousals due to abdominal discomfort.	Xie et al., 2023
Mucin degraders (e.g., *Akkermansia muciniphila*)	↓	Acetate, mucin	Gut barrier	Reduced abundance compromises the mucus layer thickness, facilitating LPS leakage and bacterial translocation.	Systemic impact: indirectly worsens all sleep parameters via enhanced inflammation.	Yuan et al., 2025

Arrows indicate the direction of change in PSI patients relative to healthy controls. BBB, blood–brain barrier; CRH, corticotropin-releasing hormone; HPA, hypothalamic–pituitary–adrenal; IDO, indoleamine 2,3-dioxygenase; IL, interleukin; LPS, lipopolysaccharide; MGB, microbiota–gut–brain; NF-κB, nuclear factor κB; PSI, post-stroke insomnia; QUIN, quinolinic acid; SCFA, short-chain fatty acid; SWS, slow-wave sleep; TLR4, Toll-like receptor 4; TRP, tryptophan; WASO, wake after sleep onset.

## 5 Therapeutic strategies: from bench to bedside

The potential reversibility of post-stroke gut dysbiosis provides a plausible therapeutic window to attenuate inflammatory signaling and improve sleep outcomes (Arya and Hu, 2018). In practice, intervention choice should be phased and individualized, because the dominant drivers (infection risk, gut barrier disruption, neuroinflammation, and sleep fragmentation) can vary across recovery stages (Zhang et al., 2024; Table 2).

**TABLE 2:** Comparative analysis of microbiota-targeted interventions.

Intervention	Key agents/targets	Mechanism (gut)	Mechanism (brain)	Best intervention phase	Safety and limitations	Study type/sample size	Key references
Psychobiotics	*Clostridium butyricum*, *Bifidobacterium longum*	Aims to support tight junctions; associated with increased butyrate production.	Hypothesized to modulate GABA/5-HT availability; may downregulate HPA axis tone via the vagus nerve.	Chronic phase (>1 month)	Safety: risk of systemic infection during the acute CIDS phase. Limit: colonization resistance (transient effect).	Meta-analysis (*N* = 11 RCTs)	Yu et al., 2024; Wang et al., 2025
Prebiotics and diet	Resistant starch, inulin, polyphenols	Selectively feeds beneficial guilds (e.g., *Bifidobacterium*, *Akkermansia*).	Boosts endogenous SCFA levels to suppress neuroglial activation.	Subacute (7 days–1 month) and chronic phase (>1 month)	Safety: generally high. Limit: bloating/gas in patients with gastroparesis.	Observational/review (N/A)	Haghighatdoost et al., 2012; Fock and Parnova, 2023
FMT/WMT	Healthy donor stool (washed preparation)	Aims to broadly restructure the microbial ecosystem; proposed to restore diversity.	May attenuate systemic inflammatory markers (e.g., IL-6, TNF-α) and potentially suppress IDO activity.	Chronic phase (>1 month, or refractory)	Safety: potential risk of aspiration pneumonia and pathogen transfer. Limit: evidence from non-stroke populations only; no PSI-specific RCTs.	Clinical study (non-stroke)/ML (*N* = 63)	He et al., 2024; Yu et al., 2025
TCM	Suanzaoren (Ziziphi spinosae semen)	Hypothesized to act as a “prebiotic-like” modulator for Bifidobacterium; may suppress pathogens.	Proposed to interact with the tryptophan-kynurenine pathway, potentially favoring serotonin synthesis.	All phases (adjuvant care)	Safety: good safety profile. Limit: herb quality control; complex pharmacology; lack of PSI-specific microbiome RCTs.	Systematic review/review (N/A)	Wu et al., 2025; Zhang et al., 2026
Acupuncture	Zusanli (ST36), sanyinjiao (SP6)	Suggested to stimulate the vagus nerve; might enhance gastrointestinal motility.	Hypothesized to signal the NTS; could activate the cholinergic anti-inflammatory pathway.	Subacute (7 days–1 month) to chronic phase (>1 month)	Safety: minimally invasive. Limit: operator-dependent efficacy; ICU logistics; lack of PSI-specific microbiome RCTs.	Review/preclinical (N/A)	Pan et al., 2025; Wu et al., 2025
Postbiotics	Heat-killed *Lactobacillus*, butyrate salts	Direct interaction with epithelial TLRs; tight junction reinforcement.	Reduces systemic inflammatory load without risk of bacterial translocation.	Acute phase (0–7 days)	Safety: excellent (no live bacteria). Limit: shorter half-life; requires precise dosing.	RCT protocol (target *N* = 208)	Franciosa et al., 2023; Corriero et al., 2025
Next-Gen probiotics	*Akkermansia muciniphila*	Mucin layer restoration; barrier fortification.	Metabolic regulation; improvement of insulin sensitivity and inflammation.	Chronic phase (>1 month)	Safety: emerging profile. Limit: difficult to culture/manufacture; regulatory hurdles.	Review (N/A)	Bao et al., 2022; Yuan et al., 2025

A strategic guide for clinical decision making. FMT, fecal microbiota transplantation; WMT, washed microbiota transplantation; TCM, traditional Chinese medicine; GABA, gamma-aminobutyric acid; 5-HT, 5-hydroxytryptamine (serotonin); HPA, hypothalamic-pituitary-adrenal; CIDS, CNS injury-induced immunodepression; RCT, randomized controlled trial; SCFA, short-chain fatty acid; IL-6, interleukin 6; TNF-α, tumor necrosis factor alpha; IDO, indoleamine 2,3-dioxygenase; ML, machine learning; NTS, nucleus tractus solitarius; ICU, intensive care unit; TLR, Toll-like receptor.

### 5.1 Psychobiotics and next-generation probiotics

The concept of “psychobiotics” was initially pioneered as a novel class of live organisms that confer mental health benefits (Dinan et al., 2013). Today, this framework has expanded. These therapeutics are typically framed as probiotic candidates selected for neuroactive or stress-relevant effects, rather than for general gastrointestinal indications alone (Lin et al., 2024). Mechanistically, selected Bifidobacterium and *Lactobacillus* strains are discussed as potential contributors to neuroactive-metabolite signaling primarily observed in non-stroke or experimental settings (including GABA-related pathways) and to gut–brain communication that can involve vagal afferent circuits (Arya and Hu, 2018; Lin et al., 2024). In the post-stroke setting, such approaches are conceptually aligned with two targets highlighted earlier: restoring inhibitory, sleep-supportive signaling and reducing inflammatory drive that can sustain hyperarousal (Lin et al., 2024). Beyond these conventional taxa, an alternative “functional guild” approach is to prioritize microbes (or consortia) that restore SCFA-related capacity, particularly butyrate-associated functions (Zhang et al., 2024). SCFAs have been linked to blood–brain barrier protection and neuroinflammatory modulation, so interventions that restore SCFA availability could plausibly address the previously described “metabolic void” driven by functional dysbiosis, rather than acting only as non-specific supplements (Fock and Parnova, 2023; Lin et al., 2024). Framed this way, the clinical goal shifts from “adding a probiotic” to selectively rebuilding depleted functions (e.g., SCFA production, barrier support, and anti-inflammatory signaling) while limiting pro-inflammatory expansion (Zhang et al., 2024).

### 5.2 Precision prebiotics and dietary modulation

Dietary modulation is a comparatively low-risk route to microbiome intervention because it aims to support endogenous communities rather than introduce exogenous strains, and it can be aligned with functional targets such as restoring SCFA-related capacity (Lin et al., 2024). Conceptually, increasing intake of fermentable substrates (e.g., resistant starches and inulin-type fibers) can support microbial fermentation and downstream SCFA availability, which is relevant because SCFAs have been linked to blood–brain barrier protection and neuroinflammatory regulation (Fock and Parnova, 2023). In rehabilitation frameworks, a “microbiome-targeted diet” can therefore be framed as a foundation strategy that is likely to require sustained adherence over weeks to months to shift ecosystem function, rather than an acute-phase rescue therapy (Zhang et al., 2024).

### 5.3 Fecal microbiota transplantation (FMT) and washed microbiota transplantation (WMT)

For a more rapid attempt at ecosystem restructuring, fecal microbiota transplantation (FMT) transfers a screened donor community to the recipient and, in principle, can deliver a broader ecological consortium than single-strain approaches (Granados-Martinez et al., 2024; Zhang et al., 2024). In stroke-relevant literature, microbiome manipulation is repeatedly discussed as a way to modify systemic inflammation and downstream neurological outcomes, which provides a rationale for considering FMT as a candidate option in carefully selected, refractory cases (Zhang et al., 2024). However, FMT also has clinically salient safety constraints (e.g., infectious transmission risk despite screening, procedure-related risks, and practical challenges in neurologically vulnerable patients), which argues for cautious positioning and rigorous monitoring rather than routine use in post-stroke insomnia pathways (Granados-Martinez et al., 2024). “Washed” or otherwise processed microbiota preparations (WMT) have been proposed as technical refinements intended to improve safety by reducing non-beneficial components while retaining viable microbes (Granados-Martinez et al., 2024). Recent clinical data suggest WMT can improve sleep quality (He et al., 2024). However, because this evidence derives from general sleep disorder populations, its direct translation to PSI remains limited pending stroke-specific RCTs. Extrapolating these findings directly to neuro-compromised stroke survivors risks over-extrapolation. In the near term, these modalities fit best as exploratory escalation therapies strictly within controlled clinical trials, rather than standard clinical practice, ideally guided by phenotyping (sleep profile, inflammatory markers, and dysbiosis pattern) to clarify who benefits and at what phase of recovery (Lin et al., 2024; Zhang et al., 2024).

### 5.4 Traditional Chinese medicine (TCM) and acupuncture

Traditional Chinese Medicine (TCM) and acupuncture serve as complementary and exploratory options for post-stroke insomnia. Their hypothesized mechanisms conceptually overlap with the microbiota–gut–brain (MGB) axis model. Preclinical work suggests that stimulating acupoints like Zusanli (ST36) could influence autonomic–immune signaling and might shift gut microbial composition in preclinical models. However, direct translational evidence in human post-stroke cohorts is extremely limited. Currently, no large-scale, PSI-specific RCTs link acupuncture- or TCM-induced microbiome changes to clinically meaningful insomnia outcomes (Arya and Hu, 2018; Zhang et al., 2024; Wu et al., 2025). Similarly, Chinese herbal medicines used for sleep (e.g., Suanzaoren/Ziziphi Spinosae Semen) are being explored as hypothetical “prebiotic-like” modulators. They are proposed to reshape dysbiosis (such as potentially increasing *Bifidobacterium* and reducing Enterobacteriaceae) and might interact with sleep-relevant metabolite pathways, including tryptophan routes. Given the current evidence base, TCM approaches should be framed strictly as a hypothesis-generating, adjunct “dual-target” concept—combining gut-directed herbal modulation with neural/autonomic modulation via acupuncture. Routine clinical adoption requires rigorous human trials incorporating microbiome, metabolomic, and validated sleep endpoints. At present, the mechanistic link between TCM or acupuncture, gut microbiota modulation, and post-stroke sleep improvement relies predominantly on indirect or general population data, highlighting an unmet need for PSI-specific RCTs to substantiate these claims (Zhang et al., 2024; Wu et al., 2025).

## 6 Translational challenges and future directions

Despite the immense potential of microbiome therapies, several critical hurdles complicate their translation from the laboratory to the standard stroke ward.

### 6.1 The safety paradox: CIDS and the “second hit”

A key translational concern is a “safety paradox”: acute stroke can be followed by CIDS, which may limit secondary immune-mediated injury but also increases susceptibility to systemic infections (Shi et al., 2018; Bietar et al., 2021). In parallel, stroke-associated gut barrier impairment and bacterial translocation have been demonstrated in experimental models, providing a biologically plausible route by which microbial products—or organisms—can access peripheral tissues during a vulnerable window (Crapser et al., 2016; Stanley et al., 2016). In this setting, the indiscriminate administration of live microorganisms in the acute phase warrants caution: although probiotics are generally safe at population level, invasive infections and bacteremia attributable to probiotic-associated organisms have been documented in susceptible, severely ill, or immunocompromised patients, and barrier disruption is repeatedly cited as a facilitating context (Kullar et al., 2023). This concern is amplified by concurrent “microbiome-active” medications commonly used in acute stroke care, which may further destabilize the ecosystem when resilience is needed most (Shi et al., 2018). Two common examples are PPIs and antibiotics. PPIs have been associated with measurable, patterned shifts in the human gut microbiome, including reduced alpha diversity (Shannon index) and depletion of key obligate anaerobe families such as Ruminococcaceae and Lachnospiraceae that include major SCFA producers (Zhang et al., 2023). Preventive (prophylactic) antibiotic strategies have also been evaluated after stroke, and large trials reviewed to date suggest reduced infection rates in some settings without consistent improvement in functional outcomes, underscoring the need to balance infection control with collateral microbiome injury (Shi et al., 2018). Accordingly, the field is increasingly interested in alternatives that aim to deliver microbial benefits without administering live organisms in the highest-risk window. “Postbiotics” have been defined by an ISAPP consensus panel as “a preparation of inanimate microorganisms and/or their components that confers a health benefit on the host,” and this category provides a framework for considering inactivated preparations or defined functional components as potentially safer options for vulnerable patients (Salminen et al., 2021).

### 6.2 The criticality of intervention timing: a phased framework

**FIGURE 2:**
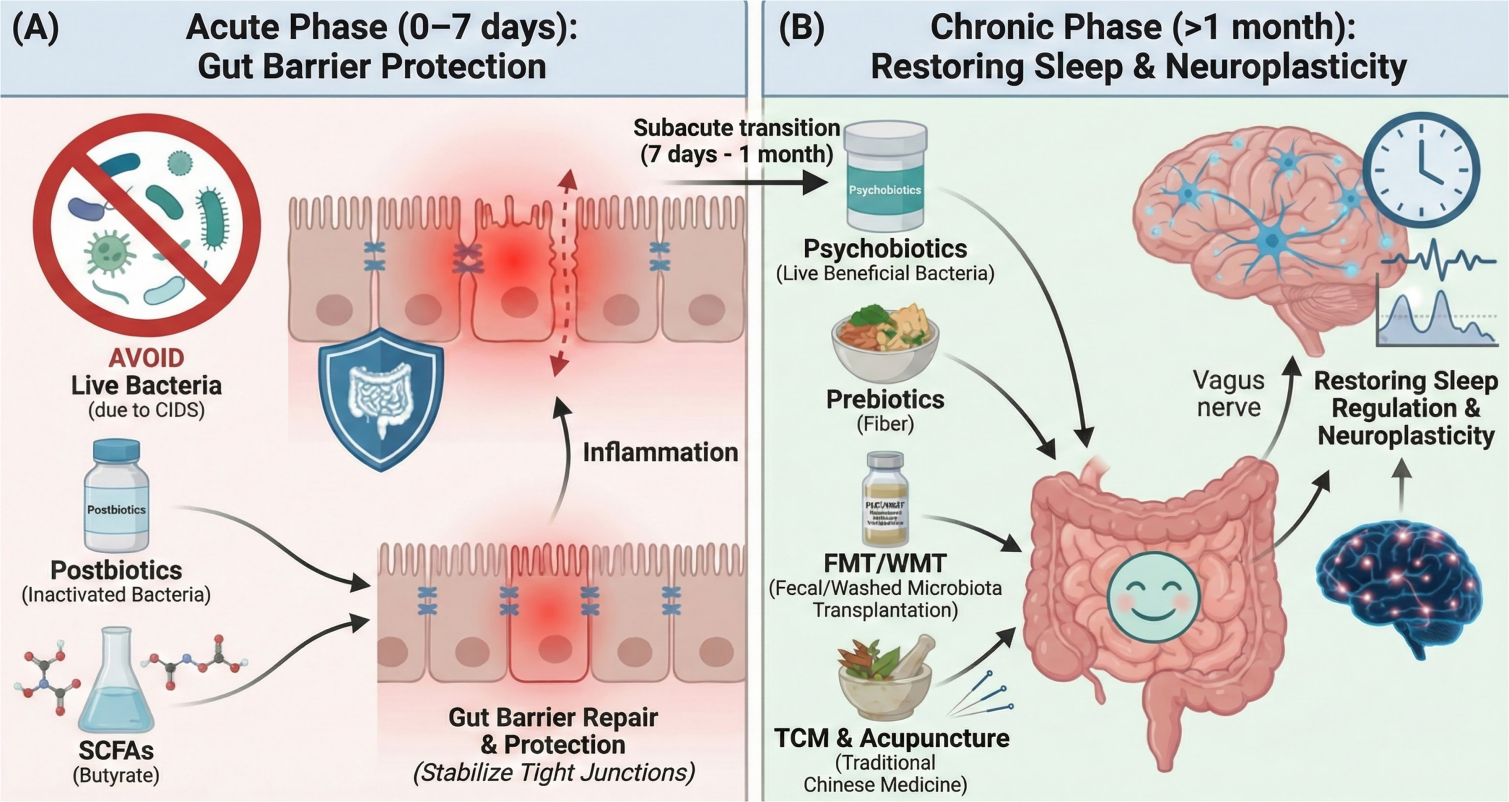
Phase-dependent therapeutic strategy. **(A**) Acute phase (0–7 days): the primary clinical goal is gut barrier protection. Due to patient vulnerability under CIDS, live bacteria are strictly avoided. Interventions focus on postbiotics (inactivated bacteria) and SCFAs (e.g., butyrate) to safely repair the intestinal wall and stabilize tight junctions. **(B)** Chronic phase (>1 month): following a subacute transition period (7 days–1 month), the therapeutic goal shifts decisively from barrier protection to restoring sleep regulation and neuroplasticity. Clinicians can safely introduce live psychobiotics, precision prebiotic diets, FMT/WMT, and TCM (including acupuncture) to support long-term sleep architecture. (CIDS, CNS injury-induced immunodepression; SCFAs, short-chain fatty acids; FMT, fecal microbiota transplantation; WMT, washed microbiota transplantation; TCM, traditional Chinese medicine).

Therapeutic success heavily relies on a strict phase-dependent approach based on the physiological evolution of stroke (Figure 2). We explicitly define the acute phase as 0–7 days post-stroke and the chronic phase as strictly beyond 1 month post-stroke. During the acute phase (0–7 days), the patient remains highly unstable with active CIDS (Figure 2A) and a collapsing gut barrier. The primary clinical goal is barrier protection, necessitating the strict avoidance of live bacteria. Interventions must focus entirely on postbiotics, short-chain fatty acids (SCFAs), and tight-junction stabilizers to safely prevent bacterial translocation. Following a subacute transition period (7 days–1 month), patient vulnerability gradually decreases. By the chronic phase (>1 month), the patient achieves medical stability (Figure 2B). Persistent insomnia, however, often stalls neuroplasticity. The therapeutic objective shifts decisively from barrier protection to metabolic restoration. Clinicians can safely introduce live psychobiotics, precision prebiotic diets, WMT, and acupuncture. These modalities effectively restore neurotransmitter networks and support long-term sleep regulation.

### 6.3 Sex as a biological variable: the “microbolome”

Accumulating data suggest that sex shapes both stroke–microbiome interactions and downstream inflammatory trajectories, which has direct implications for how post-stroke insomnia (PSI) cohorts should be analyzed and treated. In an ischemic stroke metagenomic study, women showed a distinct microbial signal involving Fusobacteriaceae, and Mendelian randomization analyses supported a potential causal contribution of higher Fusobacteriaceae levels to ischemic stroke risk, reinforcing the need for sex-stratified microbiome analyses rather than pooled inference (Lledós et al., 2023). Experimental work further supports biological plausibility for sex-linked microbiome effects on stroke recovery. In a murine ischemic stroke model with sex-cross fecal microbiota transplantation, “female-like” microbial communities were associated with lower systemic pro-inflammatory cytokines and improved survival and neurological outcomes, and the authors explicitly discuss sex hormones as likely upstream determinants of sex differences in gut microbiota and stroke susceptibility (Wang et al., 2022). Taken together, these findings justify treating sex as a core biological variable in PSI studies, with pre-specified subgroup analyses and sex-aware therapeutic design (e.g., differential targets, timing, and safety thresholds), while any hormone-adjunct strategies should be framed as hypothesis-generating until tested in controlled human trials (Wang et al., 2022; Lledós et al., 2023).

### 6.4 Precision medicine: the future of PSI therapy

The inter-individual variability of the human microbiome and its functional output makes a “one-size-fits-all” approach unlikely to be robust across stroke populations, especially when baseline dysbiosis patterns differ by sex, age, comorbidity load, diet, and medication exposures. A practical translational direction is therefore stratified intervention: using baseline stool profiling to identify (i) taxonomic states linked to inflammation risk, (ii) depletion of functional guilds (e.g., SCFA-associated communities), and (iii) metabolite patterns relevant to arousal biology, and then mapping patients to a targeted intervention class (Wang et al., 2022; Zhang et al., 2024). This approach is increasingly feasible because computational classifiers can already extract clinically relevant signals from microbiome data in post-stroke sleep-disorder contexts, supporting the use of machine learning as a triage layer for risk prediction and for selecting microbiome targets rather than applying generic supplementation (Xie et al., 2023). Methodologically, future PSI studies should also move beyond 16S rRNA profiling when possible, because shotgun metagenomics and integrative analyses (including causal inference frameworks) can better resolve strain-level and pathway-level variation that is more likely to drive treatment response than genus-level composition alone (Lledós et al., 2023).

## 7 Limitations

While the MGB axis offers a promising new frontier, current research faces several critical limitations. A primary limitation is that much of the current mechanistic framework is extrapolated from general sleep research or non-stroke animal models (e.g., MCAO). Consequently, rigorously designed, PSI-specific RCTs are urgently needed to validate these therapeutic pathways directly in human stroke survivors. These models cannot perfectly replicate the complex lifestyle, dietary, and genetic heterogeneity of human stroke patients. Clinical trials also currently suffer from small sample sizes, unstandardized probiotic dosing, and variable strain selection, yielding heterogeneous results. The direction of causality remains a complex “chicken or egg” dilemma: does dysbiosis cause insomnia, or does the stress of insomnia drive dysbiosis? Resolving this relationship requires longitudinal clinical trials with rigorous sampling protocols. Furthermore, the standardization of “Live Biotherapeutic Products” (LBPs) poses a significant regulatory hurdle. Addressing this is essential to ensure consistent product quality and clinical safety.

## 8 Conclusion

Post-stroke insomnia is not merely a distressful symptom. It is a fundamental physiological dysregulation that sabotages the brain’s ability to heal, clear toxins via the glymphatic system, and rewire itself. The converging lines of evidence reviewed herein identify the gut microbiota as a critical, yet modifiable, node in the pathology of PSI. A “vicious cycle” of autonomic dysfunction, intestinal permeability, immune activation, and metabolic deficiency (the tryptophan steal) is continuously at play. Through this cycle, gut dysbiosis perpetuates the neuroinflammation and hyperarousal characterizing insomnia. Shifting from a “brain-centric” to a “gut-brain” perspective offers a revolutionary opportunity for therapeutic intervention. Strategies ranging from dietary fiber and specific psychobiotics to washed microbiota transplantation (WMT) provide mechanism-based alternatives to traditional sedatives. They address the root cause rather than just the symptoms. Translating this to clinical application requires a nuanced, scientific approach. Clinicians must prioritize safety in the acute phase through postbiotics and embrace precision medicine in the chronic phase to tailor treatments to individual microbiome profiles. By breaking this vicious cycle, we may finally provide the injured brain with the biological foundation necessary for restorative sleep and optimal neurorehabilitation.
